# The role of the 5-hydroxytryptamine pathway in reflux-induced esophageal mucosal injury in rats

**DOI:** 10.1186/1477-7819-10-219

**Published:** 2012-10-23

**Authors:** Lingrong Yang, Haifang Cai, Jinfa Tou, Weizhong Gu, Xiaoli Shu, Ting Zhang, Xi Yang, Zheng Shen, Mizu Jiang

**Affiliations:** 1 Department of Gastroenterology, Children’s Hospital, Zhejiang University School of Medicine, Hangzhou, 310003, People’s Republic of China; 2 Key Laboratory of Reproductive Genetics (Zhejiang University), Ministry of Education, Hangzhou, 310003, People’s Republic of China; 3 The present address: The Third People's Hospital of Chengdu, Chengdu, 610031, People’s Republic of China; 4 The present address: The Central Hospital of Lishui City, Lishui, 323000, People’s Republic of China

**Keywords:** 5-hydroxytryptamine, Gastroesophageal reflux, Reflux esophagitis, 5-HT selective reuptake transporter, 5-hydroxytryptamine 4 receptor

## Abstract

**Background:**

Dysfunction of the 5-hydroxytryptamine (5-HT) signaling pathway can lead to gastrointestinal motility and secretion abnormalities and to visceral hypersensitivity. The aim of this study is to investigate the role of 5-HT in reflux-induced esophageal mucosal injury.

**Methods:**

Fifty 8-week-old male Sprague-Dawley (SD) rats were randomly divided into a gastroesophageal reflux (GER) model group (30 rats) and a sham surgery control group (20 rats). Four weeks after surgery, the esophageal mucosa was collected for histological evaluation, 5-HT concentrations, and 5-HT selective reuptake transporter (SERT) mRNA and 5-HT_4_ receptor (5-HT_4_R) protein expressions.

**Results:**

Twenty-seven rats in the GER model group survived, and three rats died. Histologically, in the GER model group, 20 rats had reflux esophagitis (RE group), and 7 rats had non-erosive reflux disease (NERD group). The 5-HT levels in the esophageal tissue from the RE group were significantly higher than those from the control and NERD groups. Both the RE and NERD groups showed significant increases in SERT mRNA expression of the esophageal mucosa than that of the controls, and the SERT mRNA level in the RE group was significantly higher than that in the NERD group. The 5-HT_4_R protein level of the esophageal mucosa in the RE group was significantly lower than that in the controls and the NERD group.

**Conclusions:**

We conclude that a 5-HT signaling pathway disorder could be a major factor in the pathogenesis of GER and RE.

## Background

Gastroesophageal reflux (GER) is defined as the reflux of the gastric contents into the esophagus or oropharyngeal area. The main etiological factors related to GER are lower esophageal sphincter (LES) dysfunction, decreased esophageal clearance capacity, esophageal mucosal barrier dysfunction, and esophageal visceral hypersensitivity [1,2]. If acidic and non-acidic substances cannot be effectively removed from the esophagus, reflux can lead to esophageal mucosal damage, causing reflux esophagitis (RE) and other complications, such as esophageal mucosal bleeding, esophageal stricture, and dysphagia. The scavenging capacity of the esophageal mucosa depends on esophageal motor function, which may play an important role in reflux-induced esophageal mucosal injury. Normal esophageal motility depends on the coordination of esophageal smooth muscle contraction and relaxation, which is the physiological basis of the normal reaction of esophageal smooth muscle to different neurotransmitters. Some studies have reported that RE is associated with impaired esophageal smooth muscle reactivity, which is a key factor in esophageal motility disorders [3,4].

It has been found that 5-hydroxytryptamine (5-HT, serotonin) signaling dysfunction can lead to gastrointestinal motility and secretion abnormalities and to visceral hypersensitivity [5,6]. 5-HT is a derivative of tryptophan and is the major precursor of 5-hydroxyindole acetic acid (5-HIAA). In humans, more than 90% of 5-HT is synthesized in enterochromaffin cells (ECs), which utilize tryptophan hydroxylase-1 (TPH-1) as their rate-limiting enzyme in the biosynthesis of 5-HT. 5-HT is released locally and acts on specific receptors located on nearby nerve fibers and cells in the lamina propria. The actions of 5-HT are terminated by 5-HT selective reuptake transporters (SERTs), which remove 5-HT from the interstitial space. Today, a total of 14 types of 5-HT receptors (5-HTRs) have been discovered. 5-HT4 receptors (5-HT_4_R) have been confirmed in human and animal esophageal tissues. The binding of 5-HT and 5-HT_4_R may activate adenylyl cyclase (AC), which increases the concentration of cyclic adenosine monophosphate (cAMP), thereby activating cAMP-dependent protein kinase A (PKA) and causing the hyperpolarization of smooth muscle cells and the reduction of Ca^2+^ influx, resulting in smooth muscle relaxation [7]. Esophageal smooth muscle relaxations play an important role in the maintenance of normal esophageal motility. Therefore, we speculated that the 5-HT signaling pathway might be involved in the regulation of esophageal motility in reflux-induced esophageal mucosal injury.

This study was undertaken by establishing a rat model of GER (as part of cardiac angioplasty), which has been shown to reflect natural GER better [8]. Esophageal pH was measured to evaluate the degree of reflux. Esophageal mucosal injury was assessed by gross findings and histopathological methods. 5-HT content and the SERT mRNA and 5-HT_4_R protein levels of esophageal tissue from rats were studied to evaluate the role of the 5-HT signaling pathway in reflux-induced esophageal mucosal injury.

## Materials and methods

### Reagents and animals

The following materials were used: 5-HT standard (Sigma, USA); polyclonal rabbit 5-HT_4_R antibody and monoclonal rabbit GAPDH antibody (Abcam, USA); HRP conjugated goat anti-rabbit secondary antibody (Zymed, USA); a SYBR PrimeScript RT-PCR kit (TaKaRa Corp., Japan); and primers for SERT and GAPDH, synthesized by Shanghai Biological Engineering Co., Ltd. (Shangon, China). Eight-week-old male SD rats were purchased from the Experimental Animal Center of Zhejiang University. All animal experiments of this study were approved by the Animal Care Committee of Zhejiang University.

## Methods

### Establishment of GER animal model

SD rats in the GER model group underwent preoperative fasting for 24 h, and no water was permitted on the morning of the day of surgery. The rats were anesthetized by an intraperitoneal injection method of 1% pentobarbital (35 mg/kg). A partial cardioplasty was performed by a longitudinal incision through the gastric cardiac muscle with reference to Erbil Y (similar to Heinecke-Mikulicz pyloroplasty) [8]. After surgery, the rats fasted for a further 48 h with free access of water. The rats that underwent sham operations served as the control group.

### Postoperative observation, determination of lower esophageal pH, and sample preparation

Following surgery, the feeding, activity, and weight change of each rat were closely observed every day. Four weeks post operation, lower esophageal pH was measured using a Digitrapper MKIII pH monitor (Synectics Medical, USA). The rat was anesthetized with an intraperitoneal injection of 1% pentobarbital (35 mg/kg). Its abdominal cavity was opened to expose the stomach and lower esophagus. Through a small hole/incision made at the greater curvature of the stomach, a pH catheter was inserted retrograde and positioned 1.5 cm above the gastroesophageal transition zone to record esophageal pH. The rats were killed, the gross findings of the esophageal mucosa were observed, and distal esophageal tissue samples were collected in several longitudinal sections. One section was fixed with 10% formaldehyde, and the others were stored at -80°C for biochemical and other studies. After creating 4-μm-thick paraffin sections, hematoxylin and eosin (HE) staining was performed for histological evaluation under an optical microscope. Reflux esophagitis (RE) was diagnosed when esophageal microscopic inflammation was observed, including basal layer thickening, vascular congestion, and eosinophil infiltration.

### Measurement of 5-HT concentrations in esophageal tissue by HPLC-ECD

Esophageal tissue was homogenized at a 1:10 weight ratio with 0.2 M perchloric acid on ice. After being centrifuged at 13,000 r/min for 30 min, the supernatant was collected and filtered with a 0.22-μm 3000D membrane (Millipore, USA) and then centrifuged at 12,000 r/min for 40 min at 4°C. The samples were then diluted two fold with 0.2 M perchloric acid and were subjected to HPLC-ECD analysis (ESA System, USA) on an ESA MD150-type C18 analytical column (3 mm × 150 mm, 3 μm) under the following conditions: electrode voltage E1 = -150 mV, E2 = 200 mV, and protective electrode voltage EGC = 350 mV. The concentrations of 5-HT in the esophageal tissue were calculated using the 5-HT standard curve.

### SERT mRNA levels detected by real-time RT-PCR

An esophageal sample of approximately 30 mg was ground into powder in a mortar using liquid nitrogen, and total RNA was extracted using the isopropanol method. cDNA was synthesized in a 10-μl system containing 2 μl of 5× Primescript™ buffer, 0.5 μl of Primescript™ RT enzyme mix I, 0.5 μl of 50 μM Oligo(dT) primer, 0.5 μl of 100 μM Random 6-mer, and 2 μl total RNA at 37°C for 15 min. The product was incubated at 85°C for 5 s and was stored at -20°C. The mRNA levels of SERT and GAPDH were detected by real-time PCR using a 7900 real-time PCR instrument (ABI, USA). The primers for SERT (forward: 5’-GATTTCCTCCTGTCCGTCATT-3’; reverse: 5’TCGGGCAGATCTTCCTCCAT-3’) and for GAPDH [forward: 5’-GACAACTTTGGCATCGTGGA-3’; reverse 5’-ATGCAGGGATGATGTTCTGG-3’, designed by Shanghai Biological Engineering Co., Ltd. (Shangon, China)] were used. The product sizes were 211 bp and 133 bp for SERT and GAPDH, respectively. The 10-μl-total reaction system contained 5 μl of 2× SYBRR Premix Ex Taq™, 0.4 μl of 10 μM PCR forward primer, 0.4 μl of 10 μM PCR reverse primer, 0.2 μl of 50× Rox reference dye and 1 μl of two-fold diluted cDNA template under the following conditions: 10 s at 95°C, followed by 40 cycles of 5 s at 95°C and 30 s at 60°C. Real-time PCR for each sample was performed in triplicate. The relative mRNA levels of the target genes to control GAPDH were calculated by ΔCt. PCR products were further examined by 1.8% agarose gel electrophoresis and were photographed using Gel Doc1000 image analysis systems (Bio-Rad, USA).

### 5-HT_4_R protein expression detected by Western blot method

Roughly 30 mg of esophageal tissue was mixed with 100 μl/10 mg lysis buffer containing fresh PMSF, and the mixture was homogenized on ice. The supernatant was collected after centrifugation at 12,000 r/min for 5 min. The protein concentration was determined using the BCA method with a SmartSpec™ protein and a nucleic acid analyzer (Bio-Rad, USA).

Protein samples of 12.5 μg were mixed with 5× sample buffer, boiled for 10 min at 95°C, and cooled on ice. After centrifugation at 12,000 r/min, the samples were subjected to SDS-PAGE and were transferred onto PVDF membrane. The membrane was then blocked with 5% BSA and was incubated with rabbit polyclonal 5-HT_4_R antibody (1:500 dilutions) for approximately 20 h on ice with oscillation. After being washed three times with TBST, the membrane was incubated with fluorescent-labeled anti-rabbit IgG at 1:5,000 dilutions at room temperature for 1 h in the dark under agitation. The band was visualized using an Odyssey dual-infrared laser system (800 nm channel, fluorescent value of 7.0). The GAPDH level was similarly detected using a rabbit monoclonal antibody (1:2,000) and the same system (800 nm channel, fluorescent value of 5.0). The images were quantitatively analyzed by converting the signals into grayscale data using Quantity One grayscale software.

### Statistical analysis

The results are expressed as the means ± standard deviations. All of the statistical analyses were performed with statistical software (SPSS, version 16.0). Student’s *t*-test or one-way ANOVA with an LSD test was used where appropriate. Significance was expressed at the *P* < 0.05 level.

## Results

### Lower esophageal pH value

Four weeks after the operation for GER, the lower esophageal pH was significantly lower in GER model group than that in controls (5.0 ± 0.28 vs. 6.4 ± 0.3, *t* = 3.757, *P* < 0.001).

**Figure 1:**
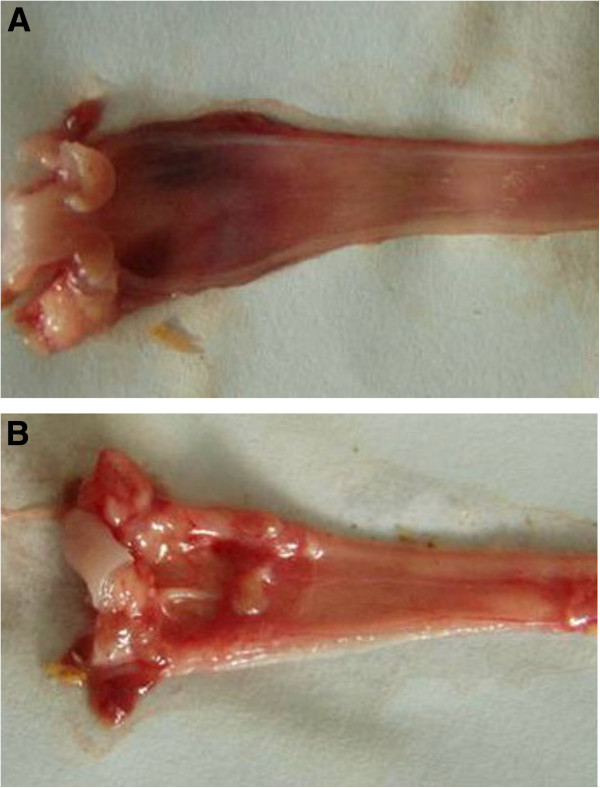
**Gross findings of esophageal mucosa in GER rat (A) and sham-operated controls (B).** Normal macroscopic esophageal mucosa is shown in **A**. The presence of gross inflammation is shown in **B**, such as esophageal mucosal congestion or erosion.

Three out of 30 (10%) rats in the GER model group died after surgery, while none of 20 rats in the control group died. As shown in Figure 1, most of the rats in the GER model group had esophageal mucosal congestion or erosion on gross examination, while there were no visible changes in controls.

### Histological assessment of the esophageal mucosa

**Figure 2:**
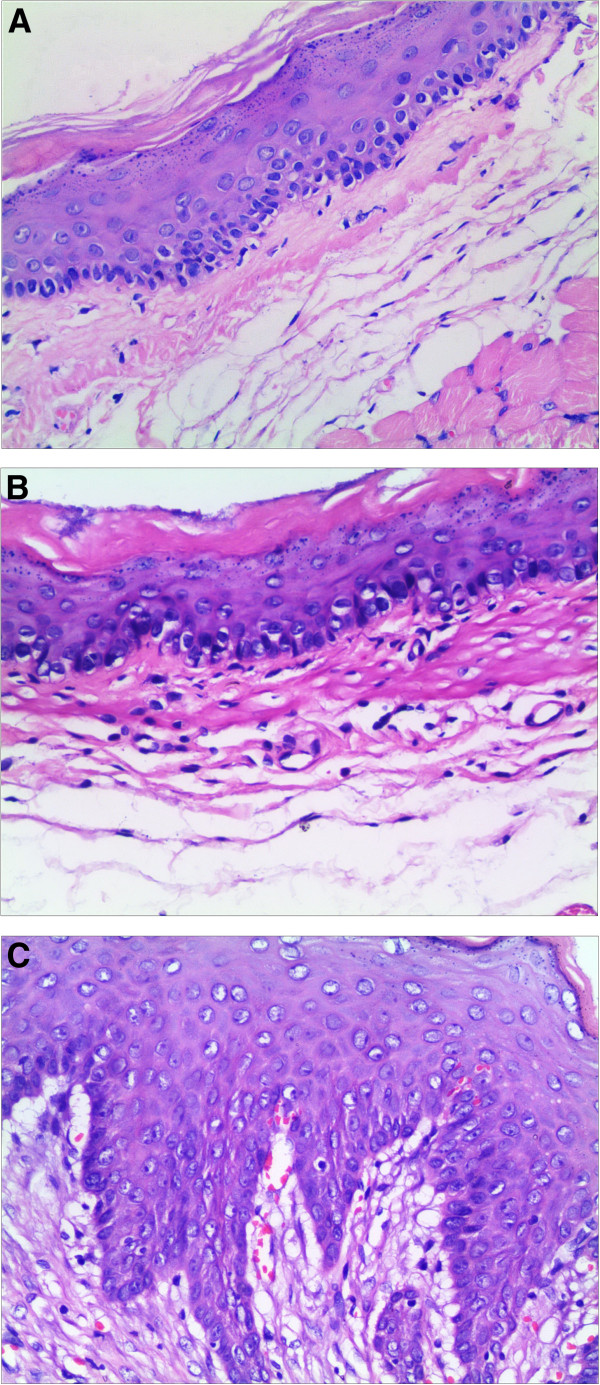
**Histological assessment of the esophageal mucosa (hematoxylin and eosin ×200) from sham operated rats (A) and GER model rats (B, C).** Normal esophageal mucosa is shown in **A** (controls). The absence of esophageal mucosal inflammation is shown in **B** (NERD). Basal layer thickening, vascular congestion, and infiltration of inflammatory cells, such as eosinophils, are shown in **C** (reflux esophagitis, RE).

Twenty out of 27 (74%) rats in the GER model group had reflux esophagitis (RE group), including basal layer thickening, vascular congestion, and eosinophil infiltration (HE ×200), while the remaining 7 rats had non-erosive reflux disease (NERD group). None of the 20 rats in the control group had inflammation or other pathological changes, as shown in Figure 2A-C.

### 5-HT content in esophageal tissue

**Figure 3:**
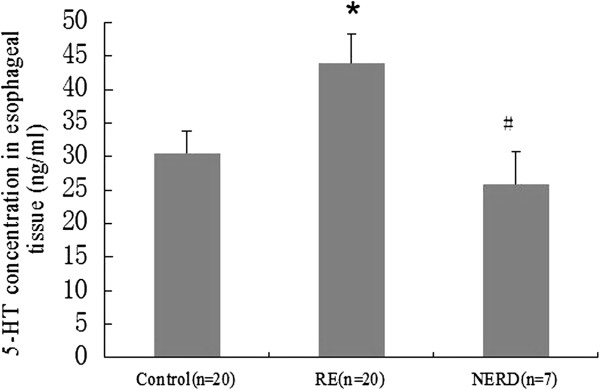
**Serotonin (5-HT) content (ng/ml) in the esophageal tissue of rats with reflux esophagitis (RE) and in NERD rats.** 5-HT levels were significantly higher in the esophageal tissue of rats with RE compared to controls (*P < 0.05). 5-HT content was significantly lower in the esophageal tissue of NERD rats than those with RE (#*P* < 0.05).

As shown in Figure 3, 5-HT levels were significantly higher in the esophageal tissue of the rats with RE than those in either the NERD or control group (*P* < 0.05).

### The relative expression of SERT mRNA in esophageal tissue

**Figure 4:**
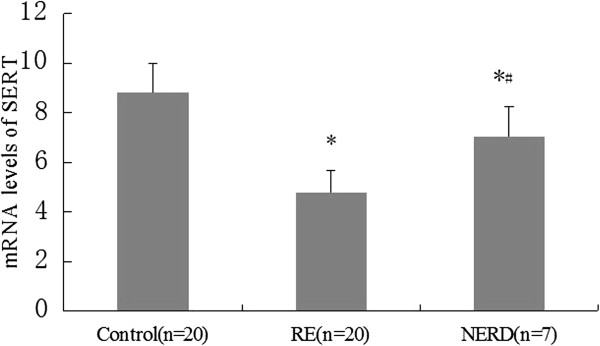
**Relative expression of serotonin reuptake transporter (SERT) mRNA levels in the esophageal tissue of rats with reflux esophagitis (RE) and NERD rats.** The results are expressed as Δct. A significantly higher level of SERT mRNA was detected in samples from both RE rats and NERD rats compared to controls (*P < 0.01), while SERT transcript levels were significantly increased in the esophageal tissue of rats with RE compared to NERD rats (#*P* < 0.01).

As shown in Figure 4, the relative expressions of SERT mRNA (Δct) in both the RE group and NE group were significantly higher than that of the controls (*P* < 0.01). The SERT transcript level in the RE group was also significantly higher than that in the NERD group (*P* < 0.01).

### The protein expression of 5-HT_4_R in esophageal tissue

**Figure 5:**
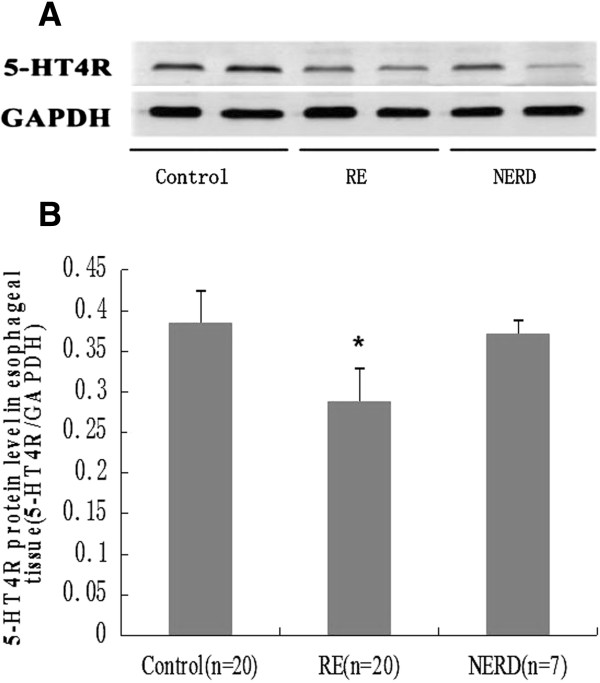
**The protein expression of 5-HT**_**4**_**R in the esophageal tissue in rats with RE and in NERD rats as detected by Western blots.** Representative Western blots of 5-HT_4_R in each group are shown at the top, with GAPDH expression used as a control (**A**). The results are expressed the ratio of 5-HT_4_R to GAPDH for immunoblots. Note that 5-HT_4_R expression is significantly downregulated in RE rats compared to NERD rats and controls. *P < 0.01 (**B**).

As shown in Figure 5, the protein expression of 5-HT_4_R in the RE group was significantly lower than that in either the NERD or control group (*P* < 0.01).

## Discussion

In addition to vomiting, GER is closely related to chronic cough, refractory asthma, recurrent pneumonia, malnutrition, and sudden infant death syndrome. However, its pathogenic mechanism is not fully understood [1,2]. Animal models are commonly used to investigate its underlying pathogenesis. In this study, we first established a GER model rat with reference to Erbil Y [8]. Our results showed that the GER model group had significantly lower esophageal pH than the sham-operated control group and that RE occurred in 74.1% (20/27) in the GER model group and in 0% in the control group, indicating that GER and RE models were successfully established.

In general, acid reflux is thought to be one of the major factors causing esophageal mucosal injury. The severity of mucosal injury depends on the nature of reflux substances, clearance ability of the esophageal mucosa, and the resistance or sensitivity of the mucosal epithelium. The clearance ability of esophageal mucosa is regulated by esophageal motor function, i.e., coordination of esophageal smooth muscle contraction and relaxation. It has been suggested that esophageal inflammation may be associated with esophageal motility disorder. It was reported that neurotransmitters regulating gastrointestinal motility, secretion, and sensation play an important role in the RE pathogenesis. Rats with esophageal reflux have decreased smooth muscle contractile responses to carbachol (receptor mediated) and KCl (non-receptor mediated), as well as decreased relaxation to 5-HT (receptor mediated) [9].

In this study, we found that the 5-HT concentrations in esophageal tissue were significantly higher in the RE group than in the controls, while the levels in the NERD group were not higher than in the controls, indicating that reflux can increase 5-HT levels in esophageal tissue. 5-HT is mainly synthesized in enterochromaffin cells through tryptophan metabolism. Synthesized 5-HT is stored as secretory granules in the basal membrane of secretory cells, functioning as paracrine molecules. Secretion of 5-HT is affected by many factors, such as local metabolites, hypertonic solution, chemical stimulation, and cell injury [10]. The increased 5-HT levels in the RE group were possibly due to acid stimulation of the esophageal mucosa. In addition, the injured esophageal mucosa perhaps further promotes 5-HT release. Released 5-HT is rapidly transported by SERT and is taken up again by adjacent cells in which 5-HT is further oxidized by monoamine oxidase into 5-HIAA and is metabolized after binding to UDP-glucuronide. Our results indicate that SERT mRNA levels in both the RE and NERD groups were significantly higher than in the controls. The SERT mRNA level in the RE group was also significantly higher than in the NERD group, suggesting that the generation of esophagitis is related to an increased SERT level. SERT can quickly transport 5-HT from cell gaps into cells, where it is inactivated, leading to decreases in esophageal peristaltic function and reflux clearance ability.

The biological function of 5-HT is exerted by binding to its specific receptor, 5-HTR. Based on the pharmacological parameters, cDNA sequence, and signal transduction mechanisms, 5-HTRs are divided into seven categories: 5-HT_1_A-E, 5-HT_2_A-C, 5-HT_3_, 5-HT_4_, 5-HT_5_, 5-HT_6_, and 5-HT_7_. Among these categories, 5-HT_1_A, 5-HT_1_B, 5-HT_1_P, 5-HT_2_A, 5-HT_2_B, 5-HT_3_, 5-HT_4_, and 5-HT_7_ are expressed in the gastrointestinal tract. 5-HT_4_R has been shown to exist in the esophageal tissues of both humans and animals [11,12]. Furthermore, activation of 5-HT_4_R can increase LES tension and promote esophageal submucosal gland (SMG) secretion [9,13]. We found that protein expression of 5-HT_4_R was significantly decreased in the RE group compared to both the NERD and control groups. 5-HT_4_R agonists, such as gastrointestinal prokinetic drugs, can promote gastrointestinal motility, accelerate clearance of reflux substrates, and reduce reflux symptoms [14]. Many 5-HT_4_R agonists can alleviate RE symptoms. For example, tegaserod, a partial 5-HT_4_R agonist, can safely and effectively reduce postprandial esophageal acid exposure by enhancing the esophageal acid clearance capacity, accelerating gastric emptying, and reducing the occurrence of transit lower esophageal sphincter relaxation (TLESR) [15], promoting bicarbonate and mucus secretion from the SMGs [13] and adjusting visceral sensitivity, thus reducing reflux, lowering the pain threshold of the esophagus to mechanical expansion, improving esophageal peristalsis, and reducing functional heartburn symptoms [14,16,17]. Another 5-HT_4_R agonist, renzapride, can promote acetylcholine (Ach) release from parasympathetic axons, increasing LES contraction and LES tension [9]. Cisapride, another 5-HT_4_R agonist, can increase LES tension and esophageal peristalsis, promoting gastric emptying, reducing the contact of gastric acid and non-acid reflux substances with the esophageal mucosa and decreasing reflux symptoms [14]. Decreased 5-HT_4_R expression, together with increased SERT expression, synergistically weakens 5-HT receptor-mediated signaling, thereby promoting GER occurrence and leading to esophageal mucosal damage. It was also reported that the SERT inhibitor citalopram can reduce esophageal sensitivity to chemical and mechanical stimuli [18].

A potential limitation of the study is related to the relatively small sample size, which did not allow us to identify the concentration of 5-HT in varying degrees of esophageal mucosal injury. Another limitation is that esophageal smooth muscle peristalsis in the RE group has not been detected. Therefore, further studies are needed to substantiate these findings.

## Conclusion

In conclusion, acid reflux may stimulate the secretion of 5-HT in the esophageal mucosa. A significant increase in SERT expression and a reduction in 5-H_4_R expression will weaken the effects of 5-HT on its effector cells, resulting in decreased post-contractional relaxation of esophageal smooth muscle, impaired coordination of contraction and relaxation, and decreased clearance of refluxed substances, subsequently causing esophageal mucosal damage and esophagitis. The results of our study suggest that the 5-HT signaling pathway plays an important role in the pathogenesis of GER.

## Abbreviations

AC: Adenylyl cyclase; cAMP: Cyclic adenosine monophosphate; ECs: Enterochromaffin cells; GER: Gastroesophageal reflux; HE: Hematoxylin and eosin; 5-HIAA: Acetic acid; 5-HT: 5-hydroxytryptamine; 5-HT_4_R: 5-HT_4_ receptor; NERD: Non-erosive reflux disease; LES: Lower esophageal sphincter; PKA: Protein kinase A; RE: Reflux esophagitis; SD: Sprague-Dawley; SMG: Submucosal gland; SERT: 5-HT selective reuptake transporters; TLESR: Transit lower esophageal sphincter relaxation; TPH-1: Tryptophan hydroxylase-1.

## Competing interests

No relevant competing financial and other interests exist for Lingrong Yang, Haifang Cai, Ting Zhang, Weizhong Gu, Jinfa Tou, Xiaoli Shu, Xi Yang, Zheng Shen, and Mizu Jiang.

## Authors’ contributions

YL contributed to the study supervision, animal study, data collection, statistical analysis, article drafting, editing, critical revision, and final approval. JM contributed to the conception and study design, study supervision, data analysis, article drafting, editing, critical revision, and final approval. YL, CH, TJ, GW, SX, ZT, YX, and SZ contributed to the GER animal model establishment, HPLC-ECD, RT-PCR, Western blotting, the esophageal histological assessment, data analysis, article editing, and final approval. All authors read and approved the final manuscript.
